# Treatment of posterolateral tibial plateau fractures: a narrative review and therapeutic strategy

**DOI:** 10.1097/JS9.0000000000001955

**Published:** 2024-07-17

**Authors:** Chen-Dong Liu, Sun-Jun Hu, Shi-Min Chang, Shou-Chao Du, Yong-Qian Chu, Yi-Ming Qi, Hao-Tao Li, Wei Mao

**Affiliations:** Department of Orthopedic Surgery, Yangpu Hospital, Tongji University School of Medicine, Shanghai, Republic of China

**Keywords:** bicondylar four-quadrant, horizontal belt plate, posterolateral tibial plateau fracture, supra-fibular head approach

## Abstract

The posterolateral tibial plateau is crucial for maintaining knee stability during flexion, and fractures in this area often involve ligament and meniscus injuries, necessitating effective management. However, treating posterolateral tibial plateau fractures (PLF) poses significant challenges due to the complex anatomy. Therefore, this review aims to explore contemporary concepts of PLF, from identification to fixation, and proposes a comprehensive treatment strategy. In this article, the authors detail the injury mechanisms, fracture morphology, PLF classification systems, surgical approaches, and techniques for open reduction and internal fixation (ORIF) as well as arthroscopic-assisted internal fixation (ARIF). The findings indicate that PLF is typically caused by flexion-valgus forces, resulting in depression or split-depression patterns. For isolated PLF, the supra-fibular head approach is often preferable, whereas posterior approaches are more suitable for combined fractures. Additionally, innovative plates, particularly the horizontal belt plate, have shown satisfactory outcomes in treating PLF. Currently, the ‘bicondylar four-quadrant’ concept is widely used for assessing and managing the tibial plateau fractures involving PLF, forming the cornerstone of the comprehensive treatment strategy. Despite challenges in surgical exposure and implant placement, ORIF remains the mainstream treatment for PLF, benefiting significantly from the supra-fibular head approach and the horizontal belt plate. Furthermore, ARIF has proven effective by providing enhanced visualization and surgical precision in managing PLF, emerging as a promising technique.

## Introduction

Highlights

- The ‘bicondylar four-quadrant’ classification system demonstrates high efficacy in assessing and managing fractures involving the posterolateral quadrant.
- The supra-fibular head approach is commonly utilized for posterolateral tibial plateau fractures, with an emphasis on avoiding neurovascular structures.
- Numerous novel plates designed for posterolateral tibial plateau fractures have been developed, notably, the ‘horizontal belt plate’ also known as the ‘hoop plate’ or ‘rim plate’, has been in widespread application.
- The advancement of arthroscopic technology has recently elevated the significance of arthroscopic-assisted internal fixation, which holds the potential to become a future trend.

Posterolateral tibial plateau fractures (PLF) are characterized as distinct articular fractures located in the posterolateral quadrant of the tibial plateau, or by a fracture line extending to the posterolateral column’s cortex^1,2^. Approximately 20% of the tibial plateau fractures involve the posterolateral quadrant, with isolated PLF accounting for about 7%^2,3^. In 1988, Waldrop *et al*.^4^ first introduced the concept of PLF and elucidated the associated injury mechanism, demonstrating that PLF often occurs when the broad convex posterior aspect of the lateral femoral condyle impacts the posterolateral tibial plateau while the knee is in a flexed position. They emphasized the primary treatment goal should be to address flexion instability.

Over the past two decades, the proliferation of low-speed vehicles, such as electric scooters, has led to an upsurge in traffic accidents, subsequently raising the incidence of PLF^1,5,6^. As research progresses, the importance of scientifically identifying and managing PLF has been increasingly recognized due to the correlation between PLF and knee flexion instability caused by bony, ligamentous, and meniscal injuries^1,4,7,8^.

The posterior tibial slope, typically around 9°, is integral to normal knee kinematics and sagittal stability. Therefore, anatomically restoring the tibial slope is crucial to prevent heightened stress on the static and dynamic knee stabilizers, the menisci, and the posterior articular surface^9^. An increasing number of studies suggest that open reduction and internal fixation (ORIF) can yield significant postoperative benefits for this fracture^1,9–11^. Besides, clinical reports have emphasized that surgical management is crucial, especially when the articular surface collapse exceeds 3 mm^12,13^. Despite the development of various approaches and fixation techniques, managing PLF presents great surgical challenges due to its risky location where it is shielded by structures such as the fibular head, lateral collateral ligaments (LCL), tendons, and critical anatomical elements like the popliteal blood vessels and common peroneal nerve situated behind the knee joint^1,6^. These complexities make the procedures of anatomical exposure and fixation placement of PLF challenging for orthopedic surgeons^1,14^.

Consequently, many scholars have endeavored to improve the management protocols, including clarifying the injury mechanism and morphological characteristics, exploring the optimal surgical approaches, developing accurate classification systems, designing the appropriate implants, and applying arthroscopic-assisted internal fixation (ARIF)^4,6,10,11,15–20^. However, these advancements have been based on different clinical cohorts rather than a unified algorithm-based recommendation. Therefore, the purpose of this paper is to examine the updated concepts and modalities of the treatment of PLF, from identification to fixation, and to provide a comprehensive treatment strategy based on our clinical experiences.

## Injury mechanism and morphological characteristics

As a distinct type of fracture, PLF often happens when individuals are riding electric scooters and relax their knees in a flexed position at ~90°. The occurrence is commonly linked to a side-fall, resulting in an axial compression force on the posterolateral plateau (flexion-valgus type violence)^9,11^. Previous studies have reported that these fractures typically manifest as depression or split-depression patterns, which often display a conical-shaped fragment, characterized by a relatively narrow articular surface area, short cortical split length, and limited depth of articular depression^1,7,16,19,21^.

The pattern, location, and degree of displacement of the fragments are influenced by the direction, magnitude, and impact point of the force, as well as the knee’s position^1,4,20–22^. In low-energy injuries, this often results in an isolated PLF without significant ligament or soft tissue injuries. However, with greater force imposing, anterior subluxation of the tibia by the femur may ensue, potentially leading to an anterior cruciate ligament (ACL) rupture^12,23^. Research findings suggest that PLF frequently leads to articular collapse and the destruction of the posterolateral cortex within a range of 30 mm^1,2,14^. In a case series involving eight patients, Chang *et al*.^1^ observed that isolated PLF had a posterior cortex length of 28 mm, with 50% of cases accompanied by fibular head fractures. Notably, those with fibular head fractures exhibited a higher articular step-off (12 mm) compared to those without (9 mm). Similarly, Gao *et al*.^2^ reported 36 cases of PLF among 242 tibial plateau fracture cases, observing that PLF had an articular step-off of 10.5 mm and a posterior cortex length of 29 mm. They also found that PLF accounted for ~14.3% of the tibial plateau’s articular surface. Additionally, Solomon *et al*.^14^ reported an articular step-off of 13.4 mm. All these findings underscore the importance of adequate interventions to achieve anatomical reduction in cases of PLF.

## PLF-related classification system

**Figure 1:**
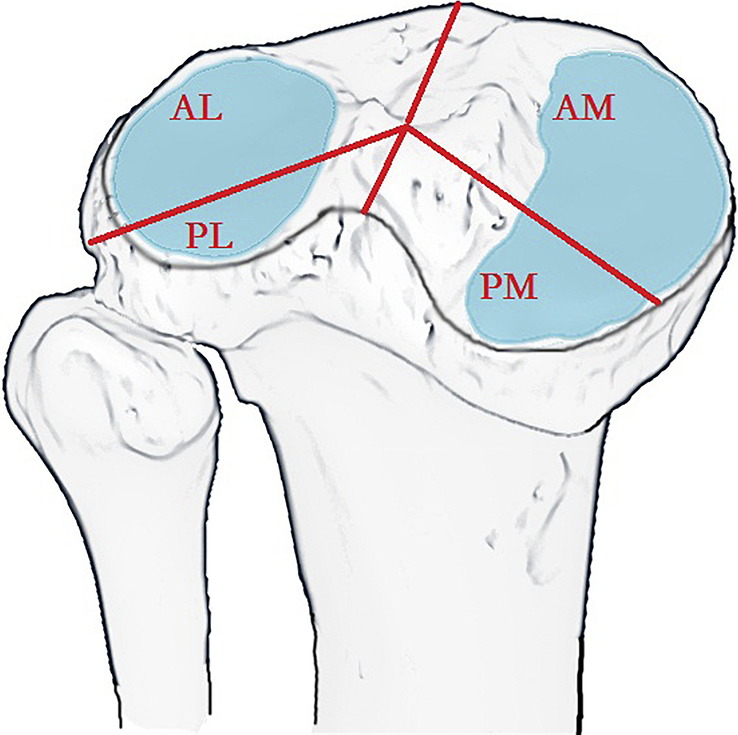
Schematic diagram of ‘bicondylar four-quadrant’ classification system. AL, anterolateral quadrant; AM, anteromedial quadrant; PL, posterolateral quadrant; PM, posteromedial quadrant. (Cited from Chang *et al*.^10^).

Currently, various classification systems exist to categorize tibial plateau fractures. Unlike earlier methods relying on X-rays, such as the Schatzker classification system, computed tomography assessment provides a more direct and practical means of identifying PLF. Luo *et al*.^24,25^ introduced a classification system based on the ‘column concept’, diverging from the traditional ‘condyle concept’, termed the ‘three-column’ approach. According to this system, fractures are classified as single-column fractures (medial, lateral, or posterior) or combined column fractures involving two or three columns, offering a novel treatment perspective of ‘column fixation’. Building upon this framework, Chang *et al*.^5,10^ highlighted that PLF could occur independently or in combination patterns. They further identified that certain central depression patterns were not discernible using previous classification systems. Additionally, the approaches addressing PLF were markedly different from those addressing the posteromedial fractures, thus prompting the proposal of a distinct classification system, called the ‘bicondylar four-quadrant’ classification system (Fig. 1). This system divides the tibial articular plateau into four quadrants, categorizing fractures based on involvement in a single quadrant (anteromedial, anterolateral, posteromedial, or posterolateral) or various combinations of two, three, or all four quadrants, emphasizing the identification of the posterolateral quadrant fractures. Moreover, research indicated that fractures within each quadrant may exhibit unique characteristics, necessitating tailored surgical approaches and interventions specific to the involved quadrant^10,15^. Presently, the ‘four-quadrant’ concept has demonstrated sufficient utility and is widely employed in the evaluation and treatment of tibial plateau fractures^15,26–28^.

In addition to the column-based and quadrant-based classifications, Zhu *et al*.^12^ conducted a retrospective review of 69 PLF cases resulting from flexion-valgus violence, categorizing them into three types. Type I fractures are characterized by depression fractures with an intact posterolateral cortex. This type is further delineated into type IA and type IB fractures, termed ‘basin-like’ fractures and ‘tongue-like’ fractures, respectively^13^. Type II fractures involve a broken posterolateral wall, presenting challenges in surgical approach selection and fixation methods compared to fractures without a broken posterolateral cortex. Type III fractures encompass PLF accompanied by an ACL injury or tibial insertion avulsion fracture of the ACL. The authors emphasized that surgical treatment is imperative for PLF with articular depressions exceeding 2 mm, particularly when coupled with an ACL injury or tibial anterior intercondylar eminence avulsion fracture.

## The common approaches

### Supra-fibular head approach

The supra-fibular head approach, one of the modified anterolateral approaches, has been frequently employed for treating PLF over the years due to its straightforward anatomy access and lack of neurovascular coverage, which significantly reduces the difficulty of surgical exposure and implant placement compared to other approaches^6^. Moreover, this approach offers the benefits of operational simplicity and secure postoperative plate removal, making it favored by many orthopedic surgeons^6,18^.

**Figure 2:**
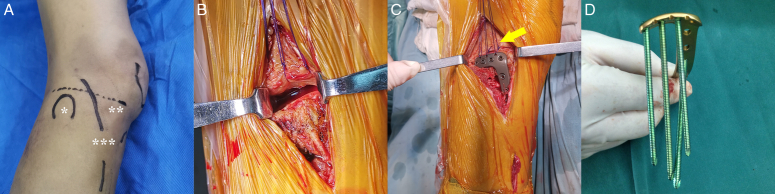
Illustration detailing the supra-fibular head approach. (A) Patient in the lateral decubitus position and incision marking. *Fibular head. **Joint line. ***Incision. (B) After coronal ligament release, the meniscus is pulled proximally by suture to expose the fractured lateral tibial articular surface. (C) Implantation of an anatomical rafting plate on the posterolateral side without dissecting any risky structures. The arrow indicates the suture knotted with the plate’s hole to restore the meniscus’s anatomical position. (D) Direct visualization of the rafting fixation effect of the anatomical rafting plate. PLF, posterolateral tibial plateau fracture.

The procedure involves several specific steps^6^. First, the patient is positioned in a lateral decubitus position with the knee joint slightly flexed to enhance posterior exposure of the articular surface. The affected knee and thigh are raised to maintain a varus position under gravity. Next, an incision is made starting from Gerdy’s tubercle on the lateral tibia, extending 3 cm upward, crossing the joint line, and encircling the fibular head, resulting in a total incision length of 10 cm. Then, the iliotibial band is dissected longitudinally, including its insertion at Gerdy’s tubercle and the soft tissue attachments on the proximal aspect of the tibia up to the lateral collateral ligament and fibular head. The coronary ligament and joint capsule are separated laterally along the lateral meniscus. After appropriate suturing, the popliteal tendon and lateral collateral ligament are retracted posteriorly by a retractor for exposure and stabilization, while the lateral meniscus is pushed proximally. Complete exposure of the posterolateral articular surface can be achieved by internally rotating the tibia (Fig. 2).

### Posterior approach

#### Posterolateral approach with osteotomy

In 1997, Lobenhoffer *et al*.^29^ first reported the fibular head osteotomy approach. This method involves making a posterolateral curved incision, dissecting along the anterior edge of the biceps femoris tendon distally to the fibular head, and then extending distally along the fibula to free the common peroneal nerve. Through the fibular neck osteotomy, the superior tibiofibular syndesmosis ligament is severed, the lateral joint capsule and the coronary ligament are incised, and the fibular head is turned upward together with the meniscus and tibial ligament to expose the articular surface of the posterolateral plateau. Fracture reduction and fixation of the PLF can be performed under direct visualization, then the fibular head is repositioned and fixed in situ.

However, this approach involves the dissociation and transfer of the common peroneal nerve and requires an osteotomy at the fibular neck, making it relatively traumatic. Solomon *et al*.^30^ extended this approach by performing an osteotomy of the fibula above the superior tibiofibular joint, completely cutting off the fibular head, and then turning it upward along with the lateral collateral ligament, allowing for wide exposure of the posterolateral area. The significant advantage of the fibular head osteotomy approach is that it provides simultaneous exposure of both the posterolateral and anterolateral tibial plateau. Several disadvantages are associated with this approach. Firstly, it requires an osteotomy procedure, which carries the risk of injuring the common peroneal nerve during surgery. Additionally, complications such as bone nonunion at the osteotomy site and the formation of pseudarthrosis may occur postoperatively.

#### Posterolateral approach without osteotomy

Carlson^31^ first introduced the direct posterolateral approach without osteotomy, which is particularly suitable for managing the isolated PLF. Typically, an 8–10 cm longitudinal skin incision is made ~1–2 cm medial to the fibular head to expose and safeguard the common peroneal nerve. Subsequently, the gap between the biceps femoris and the lateral head of the gastrocnemius muscle can be accessed, followed by peeling off the starting point of the soleus muscle to expose the posterolateral structures of the knee, ultimately positioning the plate vertically^1,32^. This approach offers the advantage of direct exposure of the fracture site, enabling reduction under direct visualization, and providing stable fixation with a supporting plate. However, it poses challenges in surgery due to the limited distal extension and the risk of injuring blood vessels, nerves, and posterolateral corner stabilizer structures.

When utilizing the traditional posterolateral approach to treat lateral bi-quadrant tibial plateau fractures, surgeons often need to change the knee’s position since it is difficult to achieve adequate exposure of the posterolateral fragments. To address this, Frosch *et al*.^3^ proposed a ‘one-incision with two-interval’ approach, containing both lateral and posterolateral incisions, aimed at increasing exposure of the entire lateral condyle. The lateral approach accesses the fracture site through the space between the anterior biceps femoris tendon and the iliotibial band, while the posterolateral approach accesses through the space between the posterior biceps femoris tendon and the lateral head of the gastrocnemius muscle.

#### Posteromedial approach

The posteromedial approach, initially developed for avulsion fractures of the posterior cruciate ligament insertion, has evolved to address various posterior knee injuries^33–35^. He *et al*.^36^ expanded its utility by successfully treating posterior tibial plateau fractures. Berber *et al*.^37^ demonstrated a modified approach to treat complex tibial plateau fractures, particularly focusing on posterior coronal shear fractures. Their surgical technique involves an inverted L-shaped as well, followed by careful manipulation of the gastrocnemius tendon to provide extensive exposure up to the posterolateral corner. This method allows for direct reduction and optimal plate positioning and offers relatively good exposure to the popliteal vessels, facilitating concurrent vascular repair when needed.

The posteromedial approach typically involves an inverted L-shaped incision behind the knee, utilizing the medial space between the medial head of the gastrocnemius and the soleus muscles to access the posterior knee structures. Advantages of this approach include the ability to expose fractures across multiple quadrants (posteromedial, posterolateral, and anteromedial) with a wide view and without the need to dissect the popliteal neurovascular bundle. Nevertheless, challenges still exist, particularly in patients with robust calf muscles where retracting the gastrocnemius muscle outwardly can be difficult, posing a risk of popliteal neurovascular bundle injury^18^.

When fixing PLF using this approach, an oblique T-plate buttress is often preferred due to its applicability for oblique placement. One drawback of the posteromedial approach is the inability to directly visualize the articular surface, requiring reliance on fragment alignment and intraoperative X-ray fluoroscopy for assessment.

## The differences between various approaches

Despite the traditional anterolateral approach being a mainstay in treating lateral tibial plateau fractures historically, it might not adequately expose the posterolateral quadrant^14,38^. Additionally, indirect reduction via a cortical window can be suboptimal, increasing the risk of secondary collapse of the articular surface. To address these issues, scholars have introduced various modifications, including adjustments to the incision location and techniques to widen the exposure area, aiming to achieve sufficient visualization of the PLF and ensure safe fixation placement^3,6,39,40^.

The supra-fibular head approach, in particular, offers a broader exposure of the posterolateral plateau with minimal risk. This approach facilitates complete visualization of the posterolateral edge of the tibial plateau and allows for the rafting of the collapsed articular surface through the fractured window itself, eliminating the need for an additional cortical window^6^. In addition, intraoperative procedures have been adjusted, such as positioning the knee joint in a flexed varus and internal rotation to increase the exposure of the posterolateral articular surface^41^. Unlike the posterior approaches, the supra-fibular head approach allows for simple plate implantation and removal due to the safe operational area, thus mitigating the limitations of the traditional anterolateral approach and rendering it now a favorable option in managing PLF^6,41^.

The deep anatomical location of PLF, obscured by the fibular head and surrounded by vital structures, including blood vessels and nerves, makes the posterior approach challenging for surgical exposure. Comparing the anatomical and intraoperative aspects of the posteromedial and posterolateral approaches, there are notable differences. First, the posterolateral approach requires meticulous avoidance of the lateral sural cutaneous nerve, originating from the common peroneal nerve, while the posteromedial incision demands caution to prevent injury to the medial sural cutaneous nerve, the great saphenous vein, and the saphenous nerve along the lower limb’s medial side. Additionally, the posterolateral approach involves entering through the lateral aspect of the gastrocnemius muscle and the soleus muscle, necessitating medial retraction of the gastrocnemius muscle and partial dissection of the popliteal muscle to visualize the articular surface. In contrast, the posteromedial approach accesses the posterior knee structures through the space between the medial gastrocnemius muscle and the soleus muscle, allowing visualization of the entire posteromedial quadrant but only a portion of the posterolateral quadrant, making it more appropriate for patients with posterior bi-quadrant fractures^34,35^.

However, limitations of the posterior approaches must be considered, including restricted longitudinal exposure imposed by the anterior tibial vascular bundle and the shielding effect of the lateral fibular head^19^. Adequate exposure of the posterolateral plateau’s articular surface may require careful dissection and stretching of the popliteal muscle or even transection of the popliteal tendon, with potential consequences on the stability of the posterolateral corner. Special attention must be given to the common peroneal nerve, which lies superficially at the fibular neck and is susceptible to injury during surgical manipulation. Additionally, the anterior tibial artery, emerging from the popliteal artery and traversing through the interosseous membrane hiatus to the anterior lower limb, poses a risk of injury during exposure and traction maneuvers.

Hu *et al*.^18^ investigated that the distance between the anterior tibial artery through the interosseous membrane hiatus and the apex of the fibula was 3.77±0.72 cm. The anterior tibial vascular bundle, composed of one artery and two concomitant veins, is usually 1 cm in width. The distances between the proximal accompanying vein and the articular surface of the tibial plateau and the apex level of the fibular head were 4.41±0.63 cm and 3.25±0.76 cm, respectively. Heidari *et al*.^36^ measured 40 specimens of lower limbs, finding that the distance from the anterior tibial artery through the interosseous membrane hiatus to the articular surface of the lateral plateau was 4.63±0.90 cm, and the distance to the fibular head was 3.57±0.90 cm. Moreover, there were four out of five cases of concomitant vein injury during the secondary plate removal operation from the posterolateral approach. This demonstrates that exposure and traction by the posterior approach are prone to injure the anterior tibial vascular bundle, especially the accompanying veins above it.

Regarding the plate placement and screw fixation, meticulous care is required to ensure proper alignment and strong fixation, with the vertical placement of the buttress plate and medial or middle screw insertion being recommended in the posterolateral approach. Preferably, 3-hole T-shaped or L-shaped buttress plates are used, and the lowest screw hole does not need to be screwed^16,18^. However, due to the coverage of the fibular head over the posterolateral plateau cortex, deviation of the plate medially during operation is a common problem. Some studies propose partial resection of the medial fibular head to enhance exposure, although this may compromise the integrity of the posterolateral corner structure and knee function^40,42^. Alternatively, the screw insertion should be directed to the lateral when using the posteromedial approach,^16,18^. Huang and Chang^19^ suggested that for patients requiring internal fixation removal, the posteromedial approach should be selected for the initial operation rather than the posterolateral approach to avoid secondary vascular injury. Notably, these issues do not arise when choosing the supra-fibular head approach^6^.

When the posterolateral approach carries risks in exposure, the posteromedial and supra-fibular head approaches offer safer alternatives with minimal risks. However, the posteromedial approach requires extensive dissection, while the supra-fibular head approach is less applicable to complex fractures involving both the posterolateral and posteromedial quadrants. Orthopedic surgeons must carefully consider the optimal surgical approach for managing these fractures. Differences between the approaches are summarized in Table 1.

**Table 1:** The differences between the common approaches for PLF.

Items	Supra-fibular-head approach	Posterolateral approach	Posteromedial approach
Incision exposure	Indirect	Direct	Indirect
Superficial structures	Iliotibial band	Lateral sural cutaneous nerve	Medial sural cutaneous nerve Saphenous nerve Great saphenous vein
Deep structures exposure	No	Lateral border of gastrocnemius-soleus	Medial border of gastrocnemius-soleus
Posterolateral articular surface	Visible	Visible	Invisible
Risk structure	No	Common peroneal nerve Anterior tibial vessels	Popliteal vessels
Plate placement	Hoop^a^ or vertical	Vertical	Oblique
Plate removal	Simple	Difficult	Moderate

^a^ Hoop means surrounding support by horizontal belt plate (hoop plate). PLF, Posterolateral tibial plateau fracture.

## The application of these approaches in specific fracture patterns

**Figure 3:**
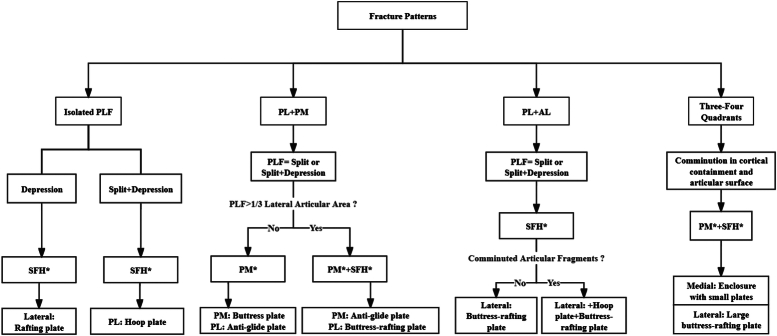
The overall treatment strategy for PLF, which provides the individualized choice of how to determine the appropriate approach and the internal fixation. AL, anterolateral; PLF, posterolateral tibial plateau fracture; PL, posterolateral; PM, posteromedial; PM*, posteromedial approach; SFH*, supra-fibular head approach.

Fractures involving the posterolateral plateau typically fall into distinct categories: isolated PLF, posterolateral plus posteromedial fractures, posterolateral plus anterolateral fractures, and three or four quadrant fractures encompassing the posterolateral plateau^10,15^. To address these varied presentations effectively, we advocate tailored treatment strategies. Drawing from our clinical expertise and contemporary research, we aimed to formulate a comprehensive treatment strategy that aligns with the ‘bicondylar four-quadrant classification system’^10,15,24,25,43^. This strategy prioritizes achieving optimal mechanical stability and favorable treatment outcomes for various types of PLF (Fig. 3). Furthermore, the illustrations of the therapeutic strategy in specific cases are presented accordingly.

### Isolated PLF

**Figure 4:**
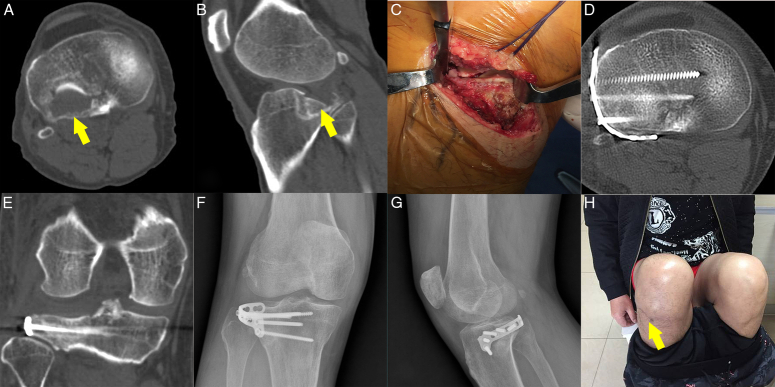
The therapeutic protocol for PLF cases. (A,B) Preoperative CT images demonstrating collapse in the posterolateral quadrant. (C) Adequate visualization of the PLF was achieved through the supra-fibular head approach. (D,E) Immediate postoperative CT images showing the collapsed articular surface effectively elevated using a horizontal belt rafting plate. (F,G) Postoperative X-rays confirm good anatomical reduction. (H) Satisfactory functional restoration was observed at the final follow-up, 12 months postoperatively. PLF, posterolateral tibial plateau fracture.

Isolated PLF commonly manifests as either a depression pattern or a combined split-depression pattern^1^. The supra-fibular head approach is suitable for managing both types of fractures. For fractures predominantly characterized by depression, the supra-fibular head approach is recommended with the lateral placement of a rafting plate. In cases of split-depression fractures, it is advisable to use a hoop plate to encircle the posterolateral cortex^1,5,6^ (Fig. 4).

### Combination fractures

#### Posterolateral plus posteromedial fractures

**Figure 5:**
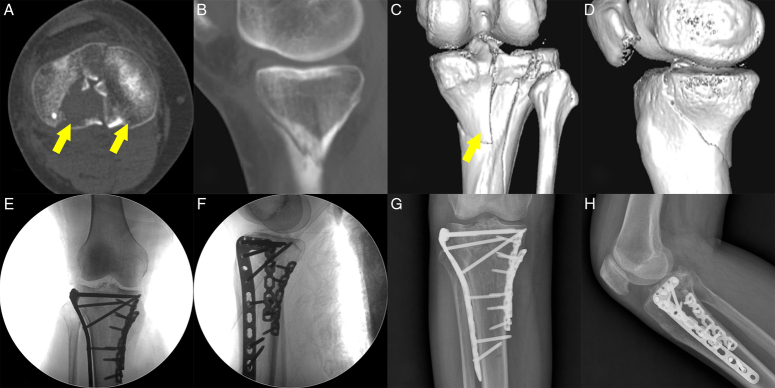
The therapeutic protocol for PL+PM fractures. (A–D) Preoperative CT images reveal a posterior bi-quadrant fracture with a PLF of more than one-third of the lateral articular area and a large posteromedial split fragment can be observed. (E,F) Rigid fixation is achieved by a lateral buttress-rafting plate to maintain the reduction of the PLF, and two reconstruction plates for the antiglide effect to stabilize the large split fragment of the PM column. (G,H) Postoperative 3-month X-rays demonstrate excellent reduction, even during flexion. PL, posterolateral; PM, posteromedial; PLF, posterolateral tibial plateau fracture.

In cases where the tibial plateau fractures manifest as posterior bi-quadrant fractures, the PLF often exhibits a split or split-depression pattern^10,44,45^. Approaches and fixation strategies are primarily determined by the extent of involvement of the posterolateral quadrant. If the posterolateral quadrant involvement is limited to one-third of the lateral articular area, an extended posteromedial approach is recommended, utilizing a posteromedial buttress plate and a posterolateral antiglide plate^31,46^. For fractures involving more than one-third of the lateral articular area, a combined approach using both posteromedial and supra-fibular head approaches may be preferable to provide adequate surgical exposure^47^. This entails fixating the posteromedial fracture with an antiglide plate and the PLF with a buttress-rafting plate^11^ (Fig. 5).

#### Posterolateral plus anterolateral fractures

**Figure 6:**
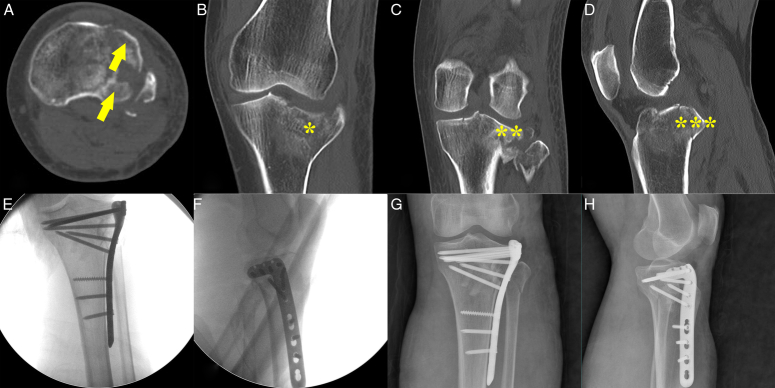
The therapeutic protocol for PL+AL fractures. (A–D) The preoperative CT scan shows the fractures affecting both the AL and PL quadrants, with instability in the posterior slope. * AL fractures. ** PL fractures. *** Posterior slope instability. (E,F) Intraoperative X-rays reveal that the articular surface of both quadrants is adequately supported by a lateral buttress-rafting plate. (G,H) Postoperative X-rays demonstrate that an excellent reduction effect has been maintained. AL, anterolateral; PL, posterolateral..

For lateral condyle fractures involving both the posterolateral and anterolateral quadrants, two surgical approaches are commonly employed: (i) supra-fibular head approach (employing a lateral rafting plate) and (ii) Frosch approach (employing a lateral rafting plate and a posterior buttress plate). Fractures of the posterolateral quadrant in this scenario typically present as split or split-depression patterns^3,27,48^. The preferred method to address this type of fracture is through the supra-fibular head approach, focusing on lateral fixation using a buttress-rafting plate with a wide horizontal limb to stabilize the entire lateral plateau. However, for fractures with comminuted articular fragments, an additional hoop plate may be necessary (Fig. 6).

#### Three or four quadrant fractures

**Figure 7:**
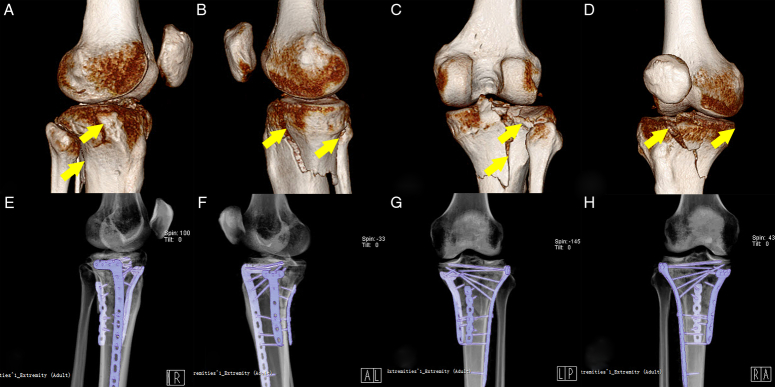
The therapeutic protocol for multiquadrant fractures. (A–D) The preoperative 3-D CT images demonstrate fractures in all four quadrants. A: AL+PL fractures. B: AM+PM fractures. C: PM+PL fractures. D: AM+PM fractures. (E–H) Postoperative volume reconstruction images show that the anatomical reduction of all four quadrants was achieved using the ‘three-column’ fixation concept. In this case, it involved using a large lateral buttress-rafting plate for overall stability, a PM buttress plate for the buttressing effect, and a PM reconstruction plate for the antiglide effect. AL, anterolateral; AM, anteromedial; PL, posterolateral; PM, posteromedial.

In cases where the tibial plateau fractures involve three or four quadrants, these fractures typically exhibit comminution involving cortical containment and the articular surface. It is recommended to use a combination of the posteromedial and supra-fibular head approaches^24,25^. The choice of implants should be tailored to the specific morphological patterns observed in each quadrant, with consideration given to the fixation of the medial, lateral, and posterior columns, respectively (referred to as ‘three-column’ fixation)^5,15,49^. To minimize implant bulk and enhance soft-tissue safety, fractures in the medial columns (posteromedial and anteromedial) are typically stabilized using small plates such as antiglide plates or buttress plates^10^. Conversely, fractures involving the lateral side often require a larger and more robust buttress-rafting plate^10,11,49^ (Fig. 7).

## Current fixation concepts and progress

### The mechanical fixation principle

In addition to selecting the appropriate surgical approach and employing precise techniques for fracture exposure and reduction, ensuring stable fixation is crucial for successful surgical outcomes. Effective internal fixation requires not only suitable implants but also techniques tailored to the fracture morphology and injury mechanism. Giordano *et al*.^50^ introduced the morphological fixation principle, categorizing PLF into two major types: split fractures and depression fractures.

For split fractures, the ‘thumb’ principle is employed, wherein the implant is positioned to exert pressure against the fracture spike, akin to pressing with one’s thumb. This technique aims to provide optimal support to the fracture, particularly the spike, thereby preventing further displacement. In contrast, for depression fractures, especially those involving disruption of the posterior cortical edge, fixation strategies should prioritize comprehensive support for the collapsed fragments. The concept underlying this principle likens the collapsed articular surface to being supported like a ‘palm’, suggesting that a horizontal belt plate should be utilized to offer both articular surface support and peripheral cortical stabilization.

### PLF-based fixation techniques

The rafting plate is a crucial technique for the treatment of collapsed PLF, typically requiring three to four screws parallel below the articular cartilage to support the articular surface^51^. Although many scholars recommend the lateral tibial rafting plates, the proximal end of most plates is relatively wide and thick, making posterior placement challenging for PLF fixation. Sassoon *et al*.^52^ found that the average extent of the posterior articular surface that could not be fixed and supported by the proximal tibial lateral locking plate was about 16 mm, encompassing almost 40% of the entire lateral quadrant. Therefore, lateral plating is usually ineffective for PLF, necessitating efforts to place the plate as far posteriorly as possible, such as the method proposed by Yu *et al*.^40^, which involves the partial excision of the fibular head to encircle the fracture.

**Figure 8:**
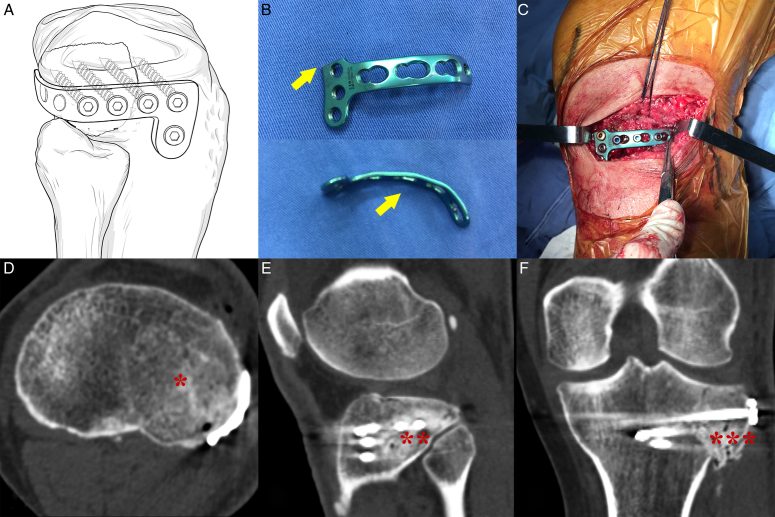
Illustration of the application of the horizontal belt plate technique. (A) A schematic drawing presents the plate location and screw insertion. (B) A 3.5 mm T-shaped plate, originally intended for distal radius fractures, is employed for PLF. Arrows indicate that the plate was bent to align with the edge of the lateral condyle (below) after one of its arms was cut off (above). (C) Using the supra-fibular head approach, the plate was positioned horizontally beneath the subchondral bone with rafting screws, following the restoration of the articular surface congruence and the insertion of the allogeneic bone graft. (D-F) Postoperative CT scans demonstrated excellent reduction of the PLF and congruence of the articular surface. * Elevated articular surface. ** Rafting effect by the horizontal belt plate. *** Allogeneic bone graft. PLF, posterolateral tibial plateau fracture.

To address these challenges, the ‘horizontal rafting plate’ technique has gradually emerged and developed rapidly. Hu *et al*.^17^ proposed using a horizontal belt plate to provide encircling fixation for isolated PLF. This method involves cutting off one arm of a 3.5 mm T-shaped plate, reshaping it into an L-shape with a 90° rotation, contouring it according to the shape of the posterolateral tibial plateau, and then securing it to the posterolateral edge with screws. Similarly, Cho *et al*.^22^ introduced the ‘rim plate’ technique to treat complex tibial plateau fractures involving the posterolateral region, utilizing the space between the LCL and the tibia. A 4-hole 2.7 mm plate was shaped according to the marginal tibial plateau and placed horizontally under the cartilage to achieve suitable fixation for the fracture, especially extending behind the lateral condyle to support the posterolateral fragment. Considering the relatively weak biomechanical stiffness of this technique and the lack of sagittal screws for stereoscopic fixation, an additional 3.5 mm anatomical plate was placed on the anterolateral side of the proximal tibia below the plate. These implants, with similar shapes and functions, are referred to as ‘horizontal belt plates’, ‘rim plates’, or ‘hoop plates’, and essentially belong to the derivation of the ‘rafting plate’. They are typically applied through the supra-fibular head approach (Fig. 8)^18,22,53–57^.

Currently, the process of implant creation is ongoing. Some Chinese scholars have designed specific plates for PLF, most of which are compatible with the anterolateral approach or the posterolateral approach, and some have undergone clinical transformation^58–61^. Based on the anterolateral approach, Ren *et al*.^61^ designed a novel plate featuring an anterior wing positioned in front of the longitudinal arm of the anatomical L-shaped plate. The locking screw inserted into the screw hole of the anterior wing can connect with the ‘hoop hook’, effectively combining the anterior wing screw and the ‘hoop hook’ as a unified structure to stabilize the posterolateral fragment. Additionally, the ‘hoop hook’ of the novel plate is relatively thin, making adjustments relatively simple if necessary. Jian *et al*.^59^ designed a posterolateral anatomical locking plate specific for the posterolateral approach and used it to treat twelve patients, achieving a mean follow-up time of 26 months with Rasmussen scores ranging from 25 to 29 and Lysholm functional scores ranging from 85 to 97. Chen *et al*.^58^ produced a posterolateral tibial plateau rotational buttress plate, which hooks the posterolateral fragment after crossing the tibiofibular hiatus and can be fixed with an anterolateral anatomical plate after reduction. Twelve patients were followed up for an average of 16.5 months, with an average knee joint range of motion of 138° at the last follow-up. Ren *et al*.^60^ introduced a novel curved support plate designed to traverse the superior tibiofibular interval via the anterolateral approach, effectively supporting the posterolateral fragments. Cai *et al*.^62^ designed an anatomical plate suitable for the supra-fibular head approach, featuring a narrow proximal end and multiple raft screws. However, many of these novel designs have not been widely adopted, and there is insufficient literature reporting on them continuously.

### Promising technique- ARIF

In recent years, the popularization of arthroscopy has significantly advanced the restoration of the articular surface and surrounding structures of the knee. Consequently, ARIF, also known as fracturoscopy, has emerged as a pivotal technique in treating PLF^63–65^. It allows for accurate reduction of the articular surface and safe screw implantation under direct visualization. Additionally, it facilitates the repair of soft tissues such as ligaments and menisci, addressing both bone and soft tissue injuries concurrently^66–68^.

A review-type investigation analyzed a total of 609 patients with a follow-up period of 52.5 months, most of whom were identified as Schatzker II and III fractures. The findings revealed that 90.5% of patients treated with ARIF had ‘good’ or ‘excellent’ outcomes, as assessed by the Rasmussen scoring system^69^. Verona *et al*.^70^ compared ARIF with ORIF in treating Schatzker I-III fractures, demonstrating that ARIF could achieve better clinical outcomes with comparable perioperative complications and radiological results. Baron *et al*.^71^ also confirmed the functional superiority of ARIF over ORIF in Schatzker I-III fractures, albeit with no significant differences in radiologic assessment and complications. Wang *et al*.^72^ evaluated the differences between ARIF and ORIF, finding that both techniques yielded satisfactory functional outcomes, but ARIF resulted in better radiological results in Schatzker I-IV fractures.

As of now, ARIF stands as a viable treatment option with numerous benefits, such as minimally invasive procedures, accurate evaluation, and superior clinical outcomes. This suggests that ARIF might become a preferred alternative for the treatment of PLF in the future.

## Conclusions

PLF can occur independently or in conjunction with fractures in other quadrants. Given the posterior plateau’s critical role in maintaining flexion stability, fractures in this region are often associated with ligament and meniscus injuries. Therefore, attention to fractures involving the posterolateral quadrant is crucial. Currently, ORIF remains the mainstream treatment and is the preferred choice for most orthopedic surgeons. However, limited surgical exposure and implant placement difficulties pose significant challenges in the treatment of PLF. Despite the development of various surgical approaches, the supra-fibular head approach offers significant advantages and should be considered the first choice, whether treating isolated PLF or multiquadrant fractures involving the PLF. Additionally, the horizontal belt plate has been confirmed to provide sufficient rafting effect, which is particularly beneficial for collapsed PLF. Furthermore, with the advancement and popularization of arthroscopic technology, ARIF has emerged as a prominent technique for treating PLF, suggesting a future trend in the management of these fractures.

## Ethical approval

This study was approved by the institutional review board of Yangpu Hospital, Tongji University School of Medicine (LL-2018-ZRKX-024 and LL-2021-WSJ-007).

## Consent

The patients reported in the manuscript signed informed consent/authorization for participation in research that included the permission to use data collected in future research projects, including the images and figure legends used in this manuscript.

## Source of funding

This article did not receive any specific grant from funding agencies in the public, commercial, or not-for-profit sectors.

## Author contribution

C.D.L., S.J.H., and S.M.C.: study concept and design; C.D.L.: drafting of the manuscript. C.D.L. and S.M.C.: acquisition, analysis, interpretation, review, and editing; C.D.L., W.M., and S.M.C.: critical revision of the manuscript. W.M., S.J.H., S.M.C., S.C.D., Y.Q.C., Y.M.Q., and H.T.L.: study supervision.

## Conflicts of interest disclosure

All authors declare that they have no conflict of interests.

## Research registration unique identifying number (UIN)

1. Name of the registry: not applicable.
2. Unique identifying number or registration ID: not applicable.
3. Hyperlink to your specific registration (must be publicly accessible and will be checked): not applicable.

## Guarantor

Shi-min Chang is the guarantor in this study.

## Data availability statement

The data or images used and/or analyzed during the current study are available from the corresponding author upon reasonable request.

## Provenance and peer review

Not commissioned, externally peer-reviewed.

## Financial support and sponsorship

None.

## Presentation

None.

## Assistance with the study

None.
